# Dietitians' perceptions of employment models used in Australian residential aged care facilities

**DOI:** 10.1111/ajag.13335

**Published:** 2024-05-30

**Authors:** Karly Bartrim, Olivia R. L. Wright, Wendy Moyle, Lauren Ball

**Affiliations:** ^1^ Centre for Community Health and Wellbeing The University of Queensland Brisbane Queensland Australia; ^2^ School of Human Movement and Nutrition Sciences The University of Queensland Brisbane Queensland Australia; ^3^ Menzies Health Institute Queensland Griffith University Gold Coast Queensland Australia; ^4^ School of Nursing and Midwifery Griffith University Nathan Queensland Australia

**Keywords:** dietitians, policy, residential aged care facility

## Abstract

**Objectives:**

The qualitative study aimed to explore dietitians' perceptions of employment status and engagement models with residential aged care facilities (RACF) and the impact on work activities and resident care.

**Methods:**

Dietitians currently working in RACF were recruited through convenience and snowball sampling, including contacting a list of dietitians who had previously consented to be contacted for research. A semi‐structured interview guide was developed by the research team, pilot‐tested and then used in each individual interview. Data were analysed using constant comparison and reflexive thematic analysis.

**Results:**

Thirty‐one dietitians (*n* = 29 female; median age, 39 years) with a range of experience working in different employment status and engagement models in RACF participated in an interview. Five themes were identified: (1) Being an employee allows for better integration and utilisation in the RACF, (2) Contract work creates a scarcity of time, (3) Ad hoc work does not meaningfully address nutrition challenges and may not be good for resident care, (4) Regularly scheduled visits support positive outcomes for residents and (5) Acknowledging many different employment models.

**Conclusion:**

Characteristics of engagement models likely affect dietitian work job satisfaction, individual resident care and food service in RACF. Regular dietetic engagement in RACFs is required to support resident‐centred evidence‐based dietetic practice and to improve residents' nutrition care. There is an opportunity for policy mandates to assist RACFs in regularly engaging a dietitian to ensure all residents have access to timely, high‐quality nutrition care.

**Policy Impact**

To support resident‐centred dietetic practice, dietitians must be well‐integrated and utilised in RACF. Both RACF and policymakers must consider ways to regularly engage dietitians to maximise residents' nutrition care.

## 1 INTRODUCTION

Dietitians have an important role in supporting older adults' health and quality of life through good nutrition.^1^ Dietitian engagement with older adults has been associated with improved nutrition status,^2^ fewer dietary deficiencies,^3^ higher protein intake^4^ and well‐nourished residents in aged care facilities.^5^ Furthermore, higher resident meal satisfaction is correlated with the availability of a food service dietitian.^5^ Dietitians collaborating with physiotherapists, occupational therapists and dentists to provide multidisciplinary nutritional support positively impacts quality of life among older adults.^6^

The integration of dietitians into residential aged care facilities (RACF) varies globally.^7^ Within Australia, the aged care sector is undergoing major reform to ensure all older adults receive high‐quality nutrition care.^8^ RACFs must now report the number of dietitian hours and funding spent on dietetic services in the Quarterly Financial Report (Food and Nutrition) provided to the Department of Health and Aged Care.^9^ The most recent data revealed that Australian RACFs engaged a dietitian for an average of .13 min per resident per day,^10^ which equates to approximately 47.45 min per resident per year. The time and engagement of dietitians in Australia are vastly different to other countries. For instance, in Ontario, Canada, there is a mandate for long‐term care homes to engage a dietitian for the equivalent of 30 min per resident per month.^11^ Other countries, such as Japan and some American provinces, are regulated by government policies that mandate regular dietitian attendance in RACFs^12^ or for a dietitian to oversee usual processes, including food preparation.^13^

A recent survey of 91 Australian dietitians working in RACF revealed that almost half attended more than five RACFs regularly, spreading their time across high volumes of residents.^14^ The survey also revealed that just over a third of dietitians attended RACFs fortnightly or monthly, indicating a scarcity of continuous availability within each facility.^14^ With the evidence that dietitians spend little time in RACFs^10^ and are often attending many RACFs infrequently,^14^ it warrants further investigation of employment status (e.g., employee or contractor) and engagement models (e.g., regular, planned or ad hoc) that dietitians utilise. Furthermore, the perceived impact that employment status and engagement models have on work activities and resident care is worth exploring.

A deeper understanding can inform recommendations to RACFs who engage dietitians for services and assist in developing policy initiatives. This study aimed to explore dietitians' perceptions of employment status and engagement models with RACFs and the impact on work activities and resident care.

## 2 METHODS

### 2.1 Overview and study design

A detailed description of the study's method has been published previously.^15^ A qualitative descriptive methodological approach^16^ was used to address the research question and to develop a deeper understanding of dietitians' perceptions of employment status and engagement models through shared examples.^17^ The methodological approach is a well‐established qualitative methodology regularly used to recognise the ‘who’, ‘what’ and ‘where’ experiences in their normal state.^17^ This manuscript includes data not previously reported. Ethics approval was obtained from the Griffith University Human Research Ethics Committee and ratified at The University of Queensland (2022, HE002096). This manuscript adheres to the Consolidated Criteria for Reporting Qualitative Research (COREQ).^18^

The research team are clinician researchers, including three dietitians and a gerontological nurse, and are all experienced with qualitative research. As the lead researcher was a RACF dietitian working in a range of employment and engagement models, there was awareness and familiarisation of the potential perceptions shared by dietitians increasing trustworthiness of the data.^19^ To minimise risk of bias, the researcher's experience was not disclosed to participants. The interviews were semi‐structured and followed an interview guide developed by the research team. The research guide was developed using the seven stages of an interview by Kvale^20^ and was pilot‐tested with three dietitians and modified based on feedback.

### 2.2 Recruitment and data collection

Dietitians working in an RACF in Australia (defined as attending an RACF for paid work within the last 2 weeks) were eligible to participate. Dietitians were recruited via convenience and snowball sampling through an expression of interest from previous work by the research team^14^ and a study flyer on social media platforms. All participating dietitians were encouraged to share the study information sheet with other dietitians, especially those with a range of experience and engagement models, to maximise variation sampling. Once dietitians were deemed eligible to participate, a mutually convenient time for an interview was arranged. Interviews were scheduled by phone or videoconference (Microsoft Teams (version 4.9 12.0 (28/7/2021) Microsoft Corporation, Washington)), and all interviews were conducted by the lead author. Interviews were automatically transcribed using the Microsoft Teams audio‐recording function and were cross‐checked by the lead researcher for accuracy. Interviews aimed to be 30–45 min long, and participants were required to provide verbal consent at the commencement of their interview. Member checking occurred by sending interview transcripts to participants, which also increased the trustworthiness of the data and ensured participants' views were accurately captured prior to data analysis. Participants received an AU$30 e‐gift voucher to recognise their contribution.

The final interview guide is shown in Table 1. Demographic data collected included age, gender, location, years working as a dietitian in RACF, employment status, experience with engagement models, hours per week working as a dietitian in an RACF role and the number of RACFs where they have a contract/are employed. Questions were primarily focused on current employment status and engagement in RACFs, including their impact on care provision. Description of employment status and engagement models are presented in Table 2.

**TABLE 1:** Interview guide used to explore dietitians' perceptions of employment statuses and engagement models with residential aged care facilities (RACF).

Research questions	Inquiry purpose	Main interview questions	Potential probing questions
What are dietitians' perceptions of different status and engagement models with RACF and the impact on work activities and resident care?	Elicit existing views of working in residential RACF with different employment statuses and/or engagement model. Provide an opportunity for open expression of views on different employment status and engagement models,	Can you describe your current working arrangements in RACF? Can you share your thoughts and experience working as a dietitian with (x) employment status and/or engagement model? Are there any other employment statuses or engagement models you have heard about or have experience with? Are there any similarities or differences in your experience carrying out dietitian activities? How does your current employment status or and/or engagement model of (x) impact how you work with residents in RACFs? Additional question‐ if the interviewee works in multiple facilities. Can you tell me about your experiences working in X aged care? Are there any similarities or differences between facilities? Does your current (x) employment status and/or engagement model benefit and support resident care? Why? Why not? Do you think the other (x) employment status and/or engagement model benefits or supports resident care? Why? Why not? Is there anything else you would like to add about your experience or perceptions of (x) employment status and/or engagement model?	Are there any insights you can share about x's employment status and/or engagement model? Do you feel your peers feel the same way about this as you? What was that like for you? You mentioned xxxx. Can you tell me more about that?

Abbreviation: X = indicates the employment status and/or engagement model described by participants.

**TABLE 2:** Description of employment status and engagement model of dietitians working in residential aged care facilities (RACF).

	Description
Employment status	
Contractor^21^	A contractor was a private practice dietitian who consulted with a RACF.
Employee^21^	An employee was defined as a dietitian who may be a staff member of the RACF, a group of RACFs governed as one organisation, or the government for government owned RACFs.
Engagement model	
Planned, regular	Planned, regular work at the RACF, such as once a week, fortnight, monthly or bi‐monthly.
Ad hoc	Ad hoc visits, also referred to as irregular hours, as needed or required and on‐call basis which included dietitians who waited for a RACF to contact them for a specific purpose such as reviewing a resident or completing a task.

### 2.3 Data analysis, rigour and trustworthiness

The NVivo program (version release 1.7 (09/2022 Lumivero, Colorado)) was used to facilitate data analysis. Transcribed data were uploaded to NVivo and analysed using constant comparison approach and six phases of reflexive thematic analysis.^22^ Concurrent with data collection, an iterative and reflective process of data analysis assisted in identifying the point at which sufficient data were collected to address the research question comprehensively.^23^ The lead researcher analysed the data independently, and the interpretation of data was frequently sense‐checked with an experienced qualitative researcher on the team. The research team regularly met for ongoing reflection and debriefing, strengthening the reflexivity, credibility and defensibility of the analytical process.^24^ The lead researcher first read and reread the transcripts to familiarise themselves with the data. The transcripts were analysed by organising the data in NVivo, and line‐by‐line coding for each question was conducted. The initial codes were converged and then placed into a table to generate prospective thematic concepts. These concepts were explored in subsequent interviews. After data collection, highly refined themes were developed using thematic coding, which included reviewing, defining and naming themes with the research team. The themes are presented with participant quotes and the participant number (Px). An audit trail for coding and categorising data was maintained throughout the data analysis.^19^ Descriptive statistics such as count and per cent were used to report the demographic data of the study participants.

## 3 RESULTS

Thirty‐one dietitians participated in an interview, the majority female (*n* = 29), with a median age of 39 years (IQR = 27, 46 years). The average interview length was 41 min, ranging from 25 to 68 min. Participants were in all states, and dietitians' years of experience working in RACF varied, with just over half (*n* = 18) incurring 0–5 years of experience and almost a quarter (*n* = 7) with 6–20 years of experience, the remainder having 21 or more years of experience (*n* = 6).

The demographic characteristics of participants are presented in Table 3. Most dietitians (*n* = 25) consulted a RACF through their work as a private practice dietitian or company, with six being employees of RACF or working within a government role attending a RACF. Most of the dietitians had experienced both regular and ad hoc engagement (*n* = 24). The number of RACFs regularly visited varied, with almost half of the participants (*n* = 13 attending between six and ten RACFs regularly). Some dietitians were unable to quantify how many RACFs they attend regularly (*n* = 5) due to the ad hoc nature of their work as an owner of a private practice or company (e.g., may assist an employee when unwell) or a unique work situation (e.g., does not attend the same RACF regularly).

**TABLE 3:** Characteristics of interview participants (*n* = 31).

Characteristics	*n*	%
Age (years)		
20–29	12	39
30–39	5	16
40–49	8	26
50–59	1	3
60–69	5	16
Gender		
Female	29	94
Male	2	6
Non‐Binary or Prefer not to say	0	0
Location		
New South Wales	7	23
Queensland	3	10
Victoria	10	32
South Australia	6	19
Western Australia	4	13
Tasmania	1	3
Years Working as a dietitian		
<1	5	16
1–5	13	42
6–20	7	23
21+	6	19
Employment status		
Contractor (*n* = 25)		
(Works for Private Practice/Company)		
Owner	7	22
Sole dietitian	(3)	
Employs other RACF dietitians	(4)	
Employee	18	58
Employee (*n* = 6)		
RACF	4	13
Government role that attends a RACF	2	6
Experience with engagement models		
Regularly	5	16
Ad hoc	2	6
Both	24	78
Hours per week working as a dietitian in RACF role		
Less than one day	5	16
2–4 days	12	39
Every day	14	45
Number of RACFs contracted/employed to		
1–5	5	16
6–10	13	42
11–20	7	23
21+	1	3
Unable to quantify	5	16

*Note*: Hours per week are hours spent as a dietitian and may not indicate onsite RACF hours.

Abbreviation: RACF, residential aged care facilities.

Five themes were identified from the data: (1) Being an employee allows for better integration and utilisation in the RACF, (2) Contract work creates a scarcity of time, (3) Ad hoc work does not meaningfully address nutrition challenges and may not be good for resident care, (4) Regularly scheduled visits support positive outcomes for residents and (5) Acknowledging many different employment models. An overview of themes and examples relating to each theme is presented in Table 4. Exemplar quotations are used to support the themes.

**TABLE 4:** Overview of themes, examples and additional quotes, exploring dietitians' perceptions of employment status and engagement models with residential aged care facilities (RACF) and its impact on work activities and resident care.

Themes	Examples	Additional Quotes
Theme 1: Being an employee allows for better integration and utilisation in the RACF	Dietitians described being an employee allowed for close involvement in RACF activities. Being an employee allows for greater integration into the RACF team including better staff collation and a team approach to nutrition care. As an employee, dietitians reported that it provides more role flexibility and autonomy. Working as an employee facilitates comprehensive, high‐quality care and allows greater ability to deliver best‐practice care to residents. Several dietitians agreed that employee status in RACFs should be standard.	*All of our staff are employed inhouse, we work from the homes and a lot of the time we also provide the support around other things happening in the home*…*and they're [the staff] a lot more willing to do things for you*. (P11) *As an employee, I found I've got a lot more flexibility in terms of what my role involved, so I could identify that there were issues. We had a lot of inappropriate referrals, so I'd make sure the criteria was [*sic*] a bit more strict. Or, we had some issues with food quality, so I'd work on the menus with the catering manager. So, I just found that I had a lot more autonomy in terms of being able to identify where the issues were and we could make improvements*. (P14) *I think the ideal world as an aged care dietitian would be that you would have an in‐house dietitian as a go to for every single aged care facility in Australia*. (P26)
Theme 2: Contract work creates a scarcity of time	Dietitians shared that working as a contractor does not support working as a team and not knowing staff can ultimately affect resident's care. Contract work was perceived to affect residents care as referrals may be missed or residents may have to wait to see a dietitian. Limited ability to make changes, which results in heavy reliance on supplements rather than changes to the diet. Dietitians described contract work harmed job satisfaction and intent to stay in their role. The biggest challenge of being in a contract is the insufficient number of allocated hours to attend a RACF. Contract work does not always allow the dietitian to work beyond the scope of an individual consultation with a resident.	*[As a contractor] it puts almost like a wall up with how much I can do. So, I feel like pretty much for those ones where I'm not there regularly, all I can kind of do is general recommendations*. (P1) *I don't know anyone; I don't know how the staff conduct their work. So, it's difficult, it's definitely difficult, and that can affect resident care*. (P23) *I've got a facility at the moment where if someone has a fall… I won't get referred until 3–4 months later when they've lost weight. If I'm seeing them three months later, I can't regain that muscle for them*. (P14) *So, I've got a home that only allows 3 hours a month…It's an hour for an initial assessment. It's 30 minutes for a review. You've given me a list of 20 people…so I'm pretty much like you either need to give me more hours or tell me which three residents you want seen*. (P8) *I would have been more heavily reliant on supplements working in a contract‐based role*. (P11) *We're not completely integrated into their [the RACF's] team*. (P30) *The big issue with being a contractor and the reason why I changed job as well because mentally that is not enough*. (P23) *As a consultant, it takes a lot longer to be able to make more long‐lasting change*. (P14)
Theme 3: Ad hoc work does not meaningfully address nutrition challenges, may not be good for resident care	Ad hoc visits heavily impact resident care, as often the work allocated does not address the root cause of the nutrition problem. Ad hoc work results in feeling poorly connected to the facility, residents, and staff. Working as an ad hoc dietitian is not supportive of resident care and is the baseline support for residents. Dietitians described low satisfaction working in an ad hoc arrangement. There are many frustrations when not seeing a resident when clinically indicated or being able to monitor the impact of nutrition care. Dietitians described feeling controlled at ad hoc RACFs and felt that the model of care is to just satisfy auditing requirements. Many dietitians mentioned advocating against ad hoc models of engagement, with many refusing to work in an ad hoc model.	*I feel like it's baseline support for the residents. It allows us to at least acknowledge if there's an issue or problem and provide some support*. (P4) *It doesn't solve the overall problem, or it doesn't really mean that you will get something positive out of your outcome if you don't get to see the resident again*. (P6) *If you're at the place more often, you're able to help the residents more in that sometimes a staff member might bump into you and say, ‘I've got a guy down here and he's got this problem with his eating, what do you think?’ Those incidental referrals are sometimes the most valuable ones for the person [resident]*. (P13) *There's a reason that I don't go back to those sites, it's just absolutely no job satisfaction*. (P29)
Theme 4: Regular scheduled visits support positive outcomes for residents	Working regularly allows dietitians to provide a holistic approach to resident care and ensures the recommendations provided will work for the RACF foodservice. Many dietitians agreed that regular visits create better outcomes for residents and allows for consistent, ongoing and timely care. Being regularly at a site allows for timely referrals from staff. Dietitians described being able to provide greater assistance to the RACF, as there are more opportunities to be involved in quality improvement projects. Regular visits allow for enhanced relationships with staff and facilitates a team approach to nutrition care. Regular care is what dietitians consider best practice, and it should be standard. Dietitians felt invested in the RACFs that allow for regular visits, and therefore, dietitians described their practice is different. Regardless of employment status (e.g., employee or contractor), it is important for dietitians to be in RACFs regularly.	*I can then actually keep tabs on people that I'm perhaps concerned about if they've had weight loss or they're not eating too well or they've got some other thing going on, I can actually then next time I'm there, I quickly check in and look up how things are going for them and see whether it might need another review from me*. (P25) *I think I really understand the whole story of the resident. Some residents, I have reviewed them since their admission … I kind of understand what strategies have been used in the past and what works, what would not work. Also, being able to have a talk with the nursing staff, listening to what is happening, what their families want and what is going on for the resident; is it a behavioural issue or a mental health issue, or it's just the food?* (P20)
Theme 5: Acknowledging there are many different employment models	Dietitians acknowledged that every RACF engages a dietitian differently, with no two of the same engagement models existing. Dietitians shared going to RACFs with both regular and ad hoc engagement models. The number of allocated hours onsite by the RACF vary. Some dietitians described the perceived benefit of different models of engagement as it provides an opportunity on what to advocate for. Regardless of employment status (e.g., employee or contractor), it is important for dietitians to be in RACFs regularly. To ensure consistency of nutrition care delivered to residents by dietitians, there needs to be a policy mandate.	*I've been in aged care dietetics for over 5 years, and I have been in every capacity you can think of: as a contracting dietitian, where I'm contracted to many different aged care facilities… and then also being internal as the senior dietitian*. (P26) *I don't think it matters whether they're employed or contracted…But just having that regular time in the facility is a way that they could definitely facilitate our role in aged care*. (P14)

### 3.1 Theme 1: Being an employee allows for better integration and utilisation in the RACF

Dietitians listed extensive perceived benefits of being an employee of a RACF. Dietitians described being an employee allows for close involvement in RACF activities and being integrated into staff teams in a way that realises their potential positive impact. Dietitians stated being an employee allowed them freedom in their role, increasing their flexibility and autonomy.

Working as an employee facilitates comprehensive, high‐quality care for residents. Dietitians associated a higher quality of work with a greater ability to deliver best‐practice care to residents. Being an employee facilitated better staff collaboration and a team approach. ‘*I find that my practice is a lot more comprehensive, and I guess you pick up on the little things a lot more. So, I would say that this is a higher quality way to work*’ (P11).

While there was overwhelming support for dietitians to work as employees, dietitians mentioned a key challenge with responsiveness to workload. ‘*Because I work full time. I guess we are all only just an e‐mail away or a phone call … So, while that's really great in some respect, you also sometimes never clock off*’ (P11).

### 3.2 Theme 2: Contract work creates a scarcity of time

Dietitians shared many challenges of working in a RACF as a contractor. Many of the issues stemmed from needing to be integrated into the RACF processes, which limits the ability to undertake activities and build strong relationships with staff and residents.

Due to poor integration in the RACF, dietitians felt that working as a contractor is not supportive of working as a team. Furthermore, dietitians expressed that working as contractors and not knowing the staff can affect resident care. Working as a contractor was thought to result in residents being missed or having to wait for care. Waiting for referrals or recommendations to be actioned and making a change beyond just seeing residents was mentioned by many dietitians. ‘*If they don't have enough hours [allocated in the contract] or they don't want to have more hours, it might just be that people get missed or they have to wait a few weeks [to receive care]*’ (P9).

As a result, dietitians described being limited in working with food service to make meaningful changes in the resident's diet, indicating they needed to prescribe more supplements than clinically indicated. The inability to make meaningful changes was further echoed by dietitians, with many dietitians mentioning the impact it had on job satisfaction and intent to stay in their role. ‘*If you're not allowed to make any changes to the menu or you're not allowed to provide staff training that's not in your contract, there is very low job satisfaction*’ (P29).

All dietitians mentioned that the most common challenge of being a contractor is insufficient allocated hours to attend a RACF, with many dietitians describing similar experiences. Dietitians agreed that contractor work does not always allow for working beyond the scope of an individual consultation with a resident. ‘*Normally the contract hours are insufficient because we provide the service more than just the dietitian consultation because we also do quite a lot of work around the food service, group education, quality improvement, malnutrition screening and all of the other activities that we know the dietitians can do as well for the home*’ (P12).

### 3.3 Theme 3: Ad hoc work does not meaningfully address nutrition challenges and may not be good for resident care

Almost all dietitians mentioned the detrimental impact of ad hoc hours on resident care, which is not beneficial to the RACF efforts nor conducive to realising dietetic care's true potential. Dietitians described that irregular visits hamper their ability to address the root cause of problems associated with nutrition and body weight, as ad hoc work often only permits working with a few residents for their clinical care. ‘*I don't feel like I've got a good grasp of what's going on there and you're going to get a snapshot of what's happened when you're actually there…You don't get to know the staff very well, you don't get to know the facility or the chef very well… I don't have that great connection or rapport with anyone at the facilities*’ (P19). Dietitians reported feeling poorly connected to the facility, residents and staff when not visiting regularly. Dietitians alluded to ad hoc work as not conducive to good nutrition care, and it is the bare minimum support for residents.

Dietitians provided many examples of the impact of ad hoc hours on a resident's care. They mentioned the challenges of delayed referrals and being able to make a meaningful change to resident's nutritional status, which was often described as a common occurrence. ‘*Whereas if it's ad hoc and just referred when there is some weight loss… you might come in when they've lost 10 kilos, whereas if you had a been there early it could have got them at 5 kilos*’ (P3).

Many dietitians described low satisfaction working through an ad hoc engagement model. They reported frustrations around being unable to review a resident when clinically indicated and being unable to monitor the impact nutrition care has on a resident. Dietitians described feeling controlled at ad hoc RACFs, with the majority suggesting that visiting ad hoc was just to satisfy auditing requirements. As such, ad hoc contracts were not advocated by dietitians, with some dietitians refusing to work in an ad hoc arrangement model for a RACF. ‘*We discourage [ad hoc visits] because a more regular visit provides a more comprehensive service and there's the ability to go in and work with food services*’ (P24).

### 3.4 Theme 4: Regularly scheduled visits support positive outcomes for residents

All dietitians agreed that regular visits to a RACF support residents' outcomes directly through individual clinical care and indirectly through working with the RACF staff and systems. Dietitians described being at a RACF regularly allows for a holistic approach to resident care and ensures the recommendations for the resident will work with the RACF food service.

Dietitians agreed regular visits create better outcomes for residents. Visiting regularly, dietitians emphasised that it allows the opportunity to provide ongoing, consistent and timely care. Beyond clinical care, dietitians could assist the RACF by continually engaging with quality improvement initiatives. ‘*I will fix the morning tea and the afternoon tea trolley to have options for the puree diet. My next step will be to implement a better high energy high protein intervention*’ (P6).

Furthermore, dietitians stated that regular contracts enhanced relationships with staff, facilitated a team approach to care and increased timely referrals. Dietitians emphasised regular care is what they consider best practice and should be standard. They recognise the impact RACFs with regular care models have. Dietitians who felt invested in the RACF stated their practice is different at that RACF. ‘*I invest into that place quite differently to what I do when I've got no idea when the next time is that I'm going to be going to either of those facilities*’ (P28).

### 3.5 Theme 5: Acknowledging there are many different employment models

Many dietitians acknowledged every RACF engaged a dietitian differently. Dietitians shared their experiences as employees or contractors and their journey of different employment statuses and engagement contracts. Most dietitians highlighted that no two of the same engagement models in RACF exist, with many dietitians providing examples of different models. For example, dietitians shared that they attend an RACF the same day and time every month (regular), but then the next RACF they have a contract with may only call when the RACF thinks the dietitian is required (ad hoc). Dietitians also mentioned the number of hours on‐site at every RACF varies.

Despite the diversity of when and how often a RACF engages a dietitian, some dietitians perceived the benefit of different engagement models. The benefit mainly stemmed from consideration of the best contract type and being able to advocate for that type. Regardless of the contract type, all dietitians agreed they must be there regularly. For consistency of resident nutrition care, dietitians felt that there needs to be a policy or engagement mandate. ‘*If we look at what dietitians need in terms of support, I think we need some sort of a mandate, some recommended hours every aged care organisation to adhere to*’ (P24).

**FIGURE 1:**
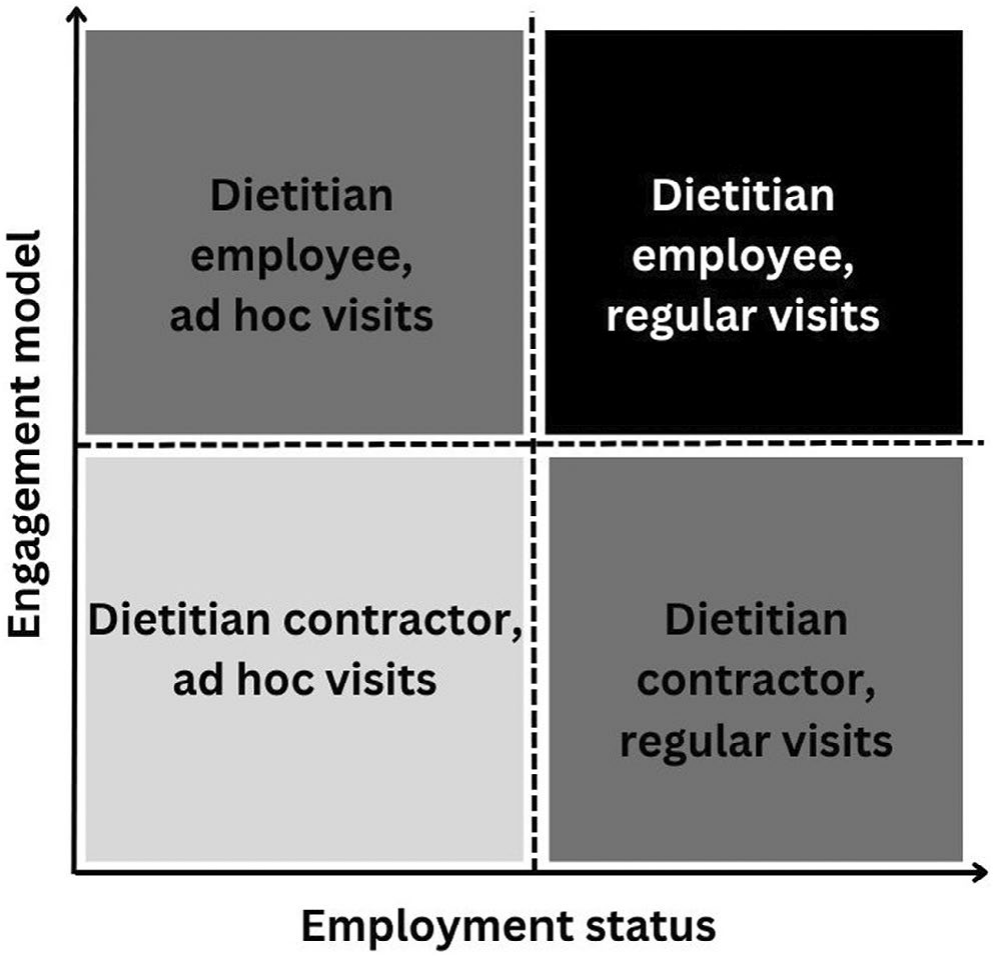
Synthesis of dietitians' views regarding engagement models. The black box highlights the preferred employment status and engagement model from dietitians of being an employee with regular visits. The grey boxes of being an employee with ad hoc visits and being a contractor with regular visits provide an alternative if employing a dietitian with regular visits is not feasible for the RACF. The grey indicates caution, as this employment status and engagement model can only be effective with mutual agreements between the dietitian and RACF, supported by best practice guidelines and resident centred‐care principles. The last box of dietitian contractors with ad hoc visits is in light grey, as it is not a recommended engagement model supporting resident care. The figure may assist RACFs when considering engaging a dietitian and other allied health professionals.

The synthesis of participants' views regarding engagement models is illustrated in Figure 1, developed from the interview themes.

## 4 DISCUSSION

This study explored dietitians' perceived impact of employment status and engagement models with RACFs and the impact on work activities and resident care. This study mirrors discussions in the United States from three decades ago regarding dietitians being involved as consultants in long‐term care.^25^, ^26^ Overall, this study aligns with recent calls for reform in RACFs to support and engage the allied health workforce better.^8^, ^27^, ^28^, ^29^ Dietitians clearly emphasise the importance of regular, scheduled engagement in RACFs and the need for policy mandates to create conditions that support high‐quality dietetic practice. Further action is required to ensure all residents in RACFs receive high‐quality nutrition care.

Irrespective of employment status and engagement models, a fundamental priority is for residents to receive high‐quality, equitable and timely care.^27^ Several dietitians reported concerns about delivering consistent and adequate nutrition care to residents working in an ad hoc arrangement, especially as contractors. Not surprisingly, dietitians felt working ad hoc with irregular visits impacts residents' care, as regular monitoring and follow‐up are essential for best‐practice nutritional management.^30^ For example, evidence‐based practice guidelines suggest that regular dietitian visits are required to manage and treat malnutrition, which affects up to 50% of RACF residents.^31^, ^32^ When dietitians do not visit residents, they can suffer significant weight loss, a key risk for malnutrition.^33^ Contract work has also been shown to negatively impact the quality of care individuals receive from hospital nurses^34^ and care staff in RACFs.^35^

Dietitians in this study emphasised their contracts or ad hoc engagements do not support the complete scope of the dietitian role or activities that could be conducted in RACF. Similar challenges of conducting activities outside direct resident care were shared by 150 long‐term care dietitians from Ontario.^36^ These challenges exist despite having an allocated 30 min per resident per month set by the Ministry of Health.^36^ As such, dietitians are advocating for greater time to complete holistic tasks such as staff education and training, quality improvement activities, participation committees, and ensuring menus meet preferences, health needs and budgets, which benefit all long‐term care stakeholders.^36^ Regular visits and adequate hours are required to deliver high‐quality care to residents and complete tasks that will assist the RACF in improving food and nutrition delivery. Future research could explore the economic and health impact on employment status and engagement models to support further changes in RACF.

The need for greater support for allied health professionals to deliver high‐quality care has also been highlighted by two recent surveys of allied health professionals working in RACFs.^28^, ^29^ Both surveys highlight that allied health professionals in RACFs are under‐resourced, delivering reduced or low‐quality and reactive care, which has been exacerbated by the COVID‐19 pandemic^28^ and more recently since the commencement of the Australian National Aged Care Classification (AN‐ACC) funding model.^29^ The present study reinforces previous findings that the allied health workforce in RACFs predominantly works on contract models part‐time and reports feeling siloed.^28^ As such, the authors of the present study support the need for the AN‐ACC funding model to include funding for allied health service provision and for the government to legislate minimum requirements for average minutes of allied health in RACF, which encompasses sufficient time for clinical care and foodservice system‐related activities.^29^ Legislation and funding changes are essential to ensure residents have access to high‐quality nutrition care from dietitians.

Ideally, dietitians should be employed by the RACF or at minimum, dietitians should have a contract that permits regular engagement with residents to provide nutrition care and allow for other activities to improve food and nutrition care delivery. The province of Ontario in Canada provides an example of how legislation mandating minutes and setting dietitian requirements (e.g., time frame to review residents and auditing the menu)^11^ could be implemented by the Australian government. To make this introduction feasible to RACFs, the government must include dietitians and the wider allied health profession in the AN‐ACC funding. Without immediate action, residents will receive ad hoc care, which may not be considered best practice or meet clinical guidelines. High rates of nutrition‐related conditions such as malnutrition will likely remain, which are not conducive to good resident health and quality of life.

The research was designed to maximise the rigour and trustworthiness of the data captured. However, there were some limitations. This study is part of a larger study that utilised a cross‐sectional design. Therefore, careful interpretation of the study's results over time must be considered due to ongoing RACF sector reforms. Another important consideration is that all dietitians, regardless of experience, were encouraged to share their perceptions despite some needing to gain direct experience working with a specific engagement model. Future research must continue exploring changes in this sector and how they may affect dietetic practice to ensure dietitians are satisfied and can deliver high‐quality care to residents.

## 5 CONCLUSIONS

Dietitians report negative implications of working in ad hoc contract models on job satisfaction, individual resident care and overall food and nutrition delivery in RACFs. Regular dietetic engagement in RACFs is required to support evidence‐based, resident‐centred dietetic practice and to improve residents' nutrition care. New legislation and dedicated funding must be considered to ensure RACFs regularly engage a dietitian so that all residents have access to timely, high‐quality nutrition care.

## FUNDING INFORMATION

A Griffith University Post Graduate Scholarship supported this research.

## CONFLICT OF INTEREST STATEMENT

No conflicts of interest declared.

## Data Availability

The data that support the findings of this study are available from the corresponding author upon reasonable request.
